# Is DIA proteomics data FAIR? Current data sharing practices, available bioinformatics infrastructure and recommendations for the future

**DOI:** 10.1002/pmic.202200014

**Published:** 2022-09-13

**Authors:** Andrew R. Jones, Eric W. Deutsch, Juan Antonio Vizcaíno

**Affiliations:** ^1^ Institute of Systems, Molecular and Integrative Biology University of Liverpool Liverpool UK; ^2^ Institute for Systems Biology Seattle Washington USA; ^3^ European Molecular Biology Laboratory EMBL‐European Bioinformatics Institute (EMBL‐EBI) Hinxton Cambridge UK

**Keywords:** data independent acquisition, data repositories, data standards, proteomics data, spectral libraries

## Abstract

Data independent acquisition (DIA) proteomics techniques have matured enormously in recent years, thanks to multiple technical developments in, for example, instrumentation and data analysis approaches. However, there are many improvements that are still possible for DIA data in the area of the FAIR (Findability, Accessibility, Interoperability and Reusability) data principles. These include more tailored data sharing practices and open data standards since public databases and data standards for proteomics were mostly designed with DDA data in mind. Here we first describe the current state of the art in the context of FAIR data for proteomics in general, and for DIA approaches in particular. For improving the current situation for DIA data, we make the following recommendations for the future: (i) development of an open data standard for spectral libraries; (ii) make mandatory the availability of the spectral libraries used in DIA experiments in ProteomeXchange resources; (iii) improve the support for DIA data in the data standards developed by the Proteomics Standards Initiative; and (iv) improve the support for DIA datasets in ProteomeXchange resources, including more tailored metadata requirements.

**Abbreviations**
API: Application Programming Interface
CV: Controlled Vocabulary
DDA: Data Dependent Acquisition
DIA: Data Independent Acquisition
FAIR: Findability, Accessibility, Interoperability and Reusability
FDR: False Discovery Rate
IDF: Identification Definition Format
MS: Mass Spectrometry
NIST: National Institute of Standards and Technology
PSI: Proteomics Standards Initiative
PTM: Post‐Translational Modification
PX: ProteomeXchange
SDRF: Sample and Data Relationship Format
USI: Universal Spectrum Identifier

## 1 INTRODUCTION

Data independent acquisition (DIA) proteomics approaches have rapidly grown in popularity in the last few years. The overarching principle is to generate fragmentation products from every peptide ion that is sampled in the MS^1^ (Mass Spectrometry) scans. DIA proteomics techniques can be further sub‐categorized into related approaches, mostly dependent upon the instrument type generating data. In some schemes, there are multiple overlapping windows of say 10–25 Daltons, as in SWATH‐MS on SCIEX TripleTOF instruments [1], on Thermo Orbitrap, and Bruker (via the “diaPASEF” method [2]), or the full mass range, as in MS^E^ and related approaches on Waters instruments. Most medium to high‐intensity precursor peptide ions generate measurable fragment ions, thus giving the possibility, in theory at least, to identify the same peptides across every MS run. The aim is to overcome the missing value problem, which can constitute one of the main drawbacks of data dependent acquisition (DDA) approaches. This should lead to more reproducible analyses and less technical variance between samples. However, DIA approaches have a different challenge: there is no direct link between a fragment ion and the precursor ion from which it was generated.

There are two main modes of DIA computational analysis: spectrum‐centric and peptide‐centric. Spectrum‐centric methods largely follow the DDA approach and attempt to generate “pseudo MS/MS spectra” where fragment ions are specifically associated with the precursor ion from which they are most likely derived. These *pseudo spectra* can then be processed like DDA data, more often through typical sequence database search tools, although the use of other DDA analysis approaches would also be possible. Peptide‐centric methods rely instead on deciding in advance which peptidoforms may be present in the sample, more often by using a spectral library, but it is also possible to use sequence databases as an input for the analysis, using software to make an in silico (predicted) spectral library [3]. Software packages can then attempt to match entries in, for example, spectral libraries to the raw data, for example, by matching the precursor mass/charge, the mass/charge (and potentially intensity) of fragmentation peaks and the normalized retention time values to infer a correct match.

For the creation of spectral libraries, we are aware of three typical paradigms. First, some labs take the samples they plan to analyze and perform a deeply fractionated DDA analysis first to create an *experimentally matched spectral library*. This mode has the advantage that retention times and fragment ion intensities should be most closely matched to the DIA data generated. The obvious downside is the cost of instrument time and that the sensitivity is ultimately limited by the restrictions of the DDA methodology. Second, there are publicly available libraries, created from a given type of samples, or simply a “pan‐species” library, assembled from multiple runs on different tissues (e.g., [4, 5]). These libraries have the advantage that for new studies, sample and instrument time do not need to be set aside for DDA runs to create a new spectral library, and public libraries may contain a wider range of the total observable peptidome. However, they have the downside that peptide retention times and fragment ion intensities will usually be less well matched to the new experiment. Third, software packages contain artificial intelligence‐trained models, which can be used to create in silico predicted spectral libraries, having learned retention times and peptide intensities from past DDA datasets [6, 7]. If the model is well trained, it can produce high‐quality libraries entries covering every possible peptide sequence. However, in practice there is likely to be a trade‐off in performance; using a public or experimental library contains peptidoforms likely to be present in the sample (e.g., particular tissues or fluids), giving better statistical power, at the expense of losing a few low abundant peptidoforms absent from a DDA library. An in silico library could be orders of magnitude bigger, and thus may give lower sensitivity of identification overall. There have only been a few benchmarking efforts to compare the different modes [8, 9], and there is no clear consensus yet.

Our focus in this Viewpoint manuscript is to consider what the rapid growth in DIA proteomics means for data sharing and standardization in proteomics. Public databases and data standards for proteomics were mostly designed with DDA proteomics in mind, and do not yet cater ideally for DIA.

### 1.1 Data sharing and FAIR data in proteomics

Since 2002, the Proteomics Standards Initiative (PSI) has developed standards covering various stages within a proteomics pipeline [10] (as well as molecular interactions [11]), including mzML for raw data or peak picked spectra [12], mzIdentML for peptide and protein identification data [13] and mzTab for a simple view of identification and quantification of peptides and proteins [14]. Various other formats and standards have been developed including the recent Universal Spectrum Identifier (USI) standard for referring to one specific spectrum and its interpretation in a public database [15]. In terms of public access to proteomics data, the ProteomeXchange (PX) consortium was established [16], originally with founder databases PRIDE and PeptideAtlas to harmonize deposition and access to proteomics data. PX expanded to include MassIVE, PanoramaPublic, iProX, and jPOST. The current situation is that a large proportion of published studies in biological journals are accompanied by data deposition into PX repositories. This has driven open science practices in the field and, as a consequence, software producers and PX resources are increasingly aligned with the FAIR data principles (Findability, Accessibility, Interoperability and Reusability [17]).

In this section, we cover how these principles are generally covered for proteomics, and then in the following section, describe some of the challenges making DIA proteomics data FAIR.

*Findability and accessibility*. Datasets submitted to any of the PX databases, can be searched and accessed from those databases via their web and in most cases, also via their programmatic interfaces like, for example, the PRIDE API (Application Programming Interface). There is also available software that can facilitate access to public datasets via these APIs (e.g., https://github.com/PRIDE‐Archive/pridepy, or the *ppx* Python package [18]). Additionally, ProteomeCentral (http://proteomecentral.proteomexchange.org/) – provides a harmonized data access portal for PX datasets from all PX resources, supporting RSS feed and advanced search mechanisms. Furthermore, PX datasets are available in other resources such as OmicsDI [19], which integrates and can be used to access public datasets from different omics approaches. It is worth highlighting that all public data in PX resources is accessible without the need of account registrations, and the data licenses are very permissive (the current default is a Creative Commons CC0 license). As a consequence, multiple sites around the world can automatically download, integrate and reprocess public datasets.

All datasets submitted to a PX repository must include raw data and the processed identification results at the very minimum (“Partial” submission). If peptide/protein identification data are provided in a PSI standard format (mzIdentML or mzTab) together with an open peak list format (e.g., mzML or mgf files), the processed results data can be parsed and linked to the corresponding mass spectra. In those cases, the dataset is considered a “Complete” submission. Complete submission is ideal since identification data is then easier to assess, re‐use, and propagate into other resources. The inclusion of the peak list formats is also useful because this captures the peak picking done. In practice, a large proportion of the submitted datasets to PX resources are partial, including the raw data in a vendor‐specific binary format, and often some non‐standardized text files containing protein identification and quantification files, such as the output text files produced by popular software such as MaxQuant.

*Interoperability*. Interoperability between software tools and resources is generally driven by the use of PSI standards, such as mzML and mzIdentML. There are multiple software (including parsers and converters) and analysis tools that support reading and/or writing PSI open formats (see e.g., https://www.psidev.info/mzML for mzML files, and https://www.psidev.info/tools‐implementing‐mzidentml for mzIdentML files). The key advantage is that PSI open data formats can be used in any operating system and/or platform.

Despite the reasonable success of PSI standards, they are still not completely adopted, for several reasons. First, mzML files are generally larger in size, sometimes much larger (for SWATH‐MS and ion mobility data) than vendor raw files, adding a significant cost in data storage and transfer times. Compression protocols for mzML files have been suggested, but none has become a widely accepted standard yet [20]. Second, if software supports vendor raw files as the expected main input, there is often not seen a particular reason to convert to mzML, since it is considered as a redundant step. This means that proteomics data is not yet, and may never be, fully interoperable, as different software packages are often designed to fulfill only a given niche, for example, supporting some technologies and vendor raw files but not others, and in some particular operating systems only.

Another factor to consider is that the majority of data in the public domain is derived from Thermo (Thermo Fisher Scientific) instruments. The Thermo API is currently available for free, and embedded into other software applications, including ProteoWizard's MSConvert [21] and ThermoRawFileParser [22], including the vendor's own routine for peak picking. In practice, this means that given a raw MS file, one could read the data and reprocess it, reproducing the results if desired, assuming the corresponding article sufficiently describes the used software parameters. However, unlike PSI formats, it is possible in the future that vendor libraries are no longer supported or do not work on some operating systems, meaning that raw files cannot be guaranteed as accessible in the future. For data from other instrument vendors, the situation is often less straightforward. Peak picking for raw data from other instrument vendors is not always available, and thus in these cases, a Partial submission is much less useful and data may not in fact be “accessible,” even if deposited in PX. It is worth noting that SCIEX provides support for peak picking in their own converter (https://sciex.com/support/software‐support/software‐downloads), but it works in Windows only. For DIA data, the situation is yet more complex, and addressed in a later section.

Another key point is the use of controlled vocabularies (CVs) and ontologies for enabling the interoperability of metadata between tools and databases [23]. The PSI has developed several CVs (which are updated continuously) for supporting the open formats, including, for example, the PSI‐MS [24] (for all types of MS‐related data), and PSI‐MOD [25] (for protein modification data).

*Reusability*. The large growth of datasets available in PX has led to a significant amount of data reuse and repurposing, including, for example, software benchmarking efforts, “big data” approaches that make use of artificial intelligence techniques [26], proteogenomics (finding MS evidence to support annotation of gene models as coding) [27, 28], creation of tissue atlases [29 ^,^ 30] and discovery and annotation of post‐translational modifications (PTMs) [31, 32], among others. Most reuse of proteomics data is based on the reprocessing of DDA data since this has been the dominant approach with large volumes of data in PX, and they are easiest to reprocess using freely available popular software.

### 1.2 Other existing bottlenecks

There are additional bottlenecks preventing proteomics data and tools becoming more “FAIR.” This applies to both DDA and DIA approaches. First of all, regrettably, the field has not yet agreed on the adoption of an open standard format that can be used to encode quantification data (i.e., expression matrixes). The mzTab format was originally developed with this idea in mind, but it has been mostly used so far for identification data only, and it has some shortcomings.

In the context of data sharing, historically there has been very limited (standardized) sample metadata and experimental design information in PX resources. When PX was established, the focus was put on making data sharing popular in the field, so the requirements in this context were not very high. Only recently, the MAGE‐TAB‐Proteomics format has been developed to enable a standard encoding of sample metadata in public datasets, including the experimental design [33]. The format has two components: (i) the Identification Definition Format (IDF), which contains information at the level of the dataset (data already available in all PX datasets); and (ii) the SDRF (Sample and Data Relationship Format)‐Proteomics file, which contains the mappings between the raw files and samples. As of August 2022, around 450 datasets have already this type of information in PRIDE. However, for the submission of SDRF‐Proteomics files to become mandatory, it will be required that popular software tools support and export the format.

### 1.3 What does FAIR data mean for DIA proteomics?

**FIGURE 1:**
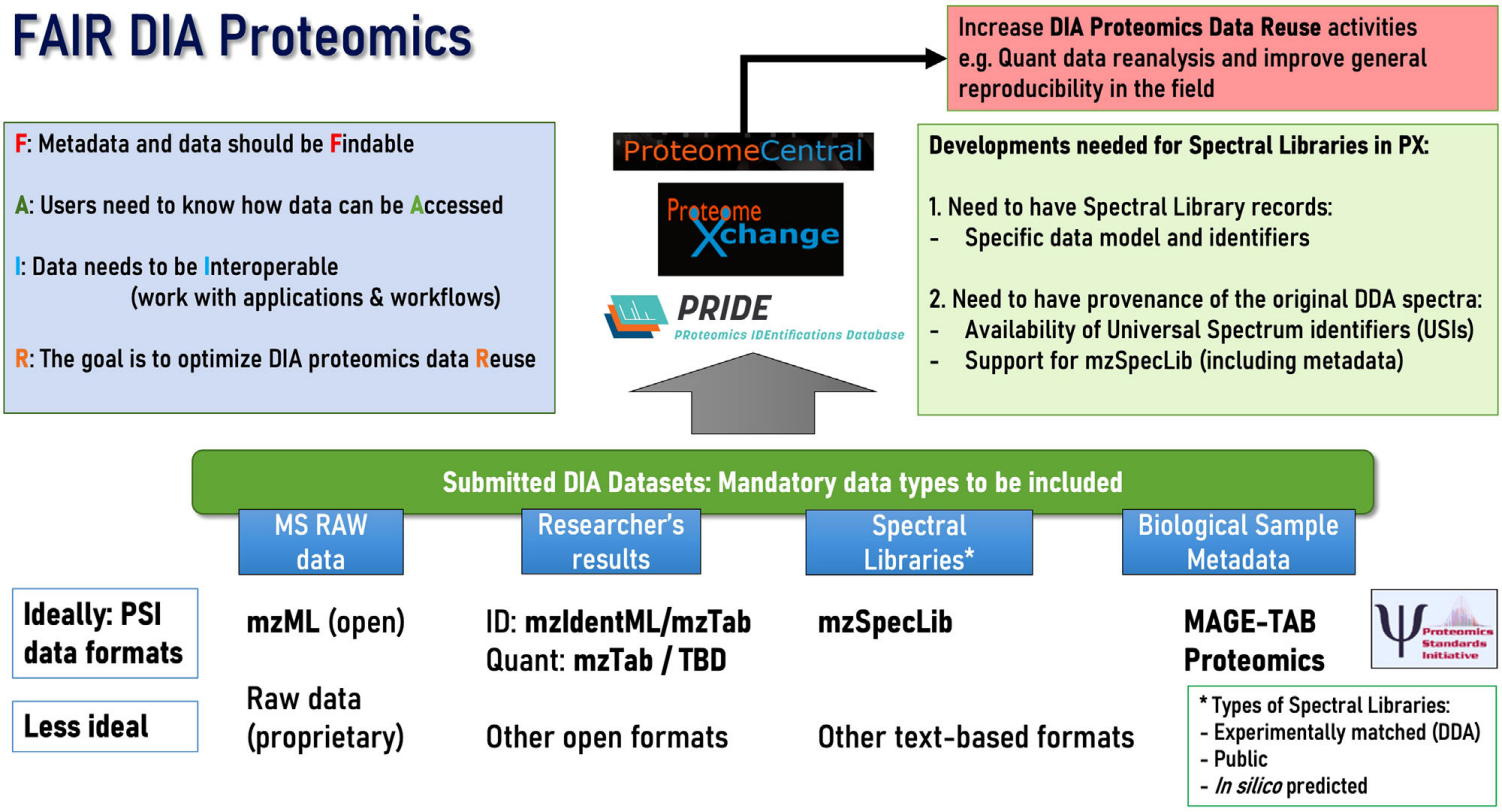
Graphical summary of the issues and recommendations related to DIA proteomics

There are some aspects of DIA proteomics data that are unique, and provide extra challenges for data sharing, and making data FAIR. Here we describe aspects of “FAIRness” for DIA data, as summarized in Figure 1.

*Findability*. At present, metadata and hence search facilities at PX databases do not easily record the width of the fragmentation window – the key parameter governing whether this can be viewed as a DIA or DDA approach. If a user wished to find all DIA datasets on a given sample type or species in all PX resources, it would not be straightforward to locate these datasets, without trawling through lots of records and reading associated manuscripts. In PRIDE it is possible to label all submitted datasets using different sub‐types of DIA approaches (e.g. SWATH‐MS, MS^E^, etc) but this labeling needs to be standardized between all PX resources, and also the information be accessible via ProteomeCentral.

*Accessibility and reusability*. DIA data is being submitted to PX, using the same mechanism of “Partial” or “Complete” submissions. In practice, most submissions are “Partial” at present, since it would be challenging to create sensible mzIdentML/mzTab files, which were mostly designed with sequence database search methods in mind. It should be noted that MaxQuant (from version 2.0) can export mzTab coming from both DDA and DIA approaches, but so far contains identification information only [34].

Many SWATH‐MS datasets often do get converted to mzML when processed with popular software such as OpenSWATH [35], and MSConvert can generate valid mzML files for DIA data just as well as for DDA data. However, SWATH‐MS data is also commonly processed with the SCIEX Vendor software PeakView. Via this route, PX submitted datasets tend to include raw data in SCIEX “wiff” and “scan” formats, which are generally not natively supported by open‐source applications. Similarly, labs performing DIA analysis via the Bruker diaPASEF technology, generate and analyze data from the Bruker “.d” folder file format, and most current PX diaPASEF submitted datasets have deposited data in this raw format. There is support for wiff and “.d” formats in MSConvert (although as noted above, depending on the age of the instrument, we believe “vendor” peak picking does not always function in MSConvert), and natively in some free DIA analysis software, such as DIA‐NN [36] and commercial software such as Spectronaut (Biognosis). The Thermo DIA technologies use the same raw file format and APIs as in DDA, so open‐source support is quite good. This means that currently deposited raw data can usually be opened, visualized, and in theory, reprocessed. However, there are some major holes in the current data‐sharing landscape for DIA proteomics, which make this very challenging in practice.

As explained above, in most DIA approaches, a spectral library is used for identification and assignment of peaks within raw data to peptidoforms, although pseudo‐spectra are sometimes used. For the pseudo‐spectra approach, the generated peak list could be converted to mzML (or a simple open format like MGF), and submitted to PX, but in practice, this is rarely, if ever done. For data types such as Waters MS^E^, a person interested in performing data reuse would likely need some commercial vendor software to easily reprocess the data. For spectral library‐based approaches, data reuse is possible but reproducibility is dependent on the availability of the actual spectral library used in the original study. Spectral libraries are sometimes sourced from an existing repository of libraries, such as SWATHAtlas [37] (http://www.swathatlas.org/), which contains spectral libraries in open text‐based formats including some metadata describing how the library was generated. Importantly, SWATHAtlas also references the source of the DDA data used to generate the library.

A key component of the evidence trail is that some peaks within DIA data have been matched to an entry in a spectral library, working under the assumption that the library entry has been annotated with the correct peptidoform. If the library entry is incorrectly annotated, for example, because of inadequate statistical control when processing DDA data, the use of a poorly tailored theoretical proteome, or worse, by a deliberate manipulation (in potential cases of scientific fraud), then quantitative data assigned to a given peptidoform and protein, will be incorrect. It is then essential that a full evidence trail back to the source of library entry is also provided. However, the existing data formats for spectral libraries do not have a standard method for representing the actual source spectrum. Worse, most library formats created from experimentally matched DDA data, do not contain any reference back to the spectra themselves. There are only a handful of current PX datasets that contain both DIA data and the DDA data on which the library was generated. However, even in those cases, it is common practice that either the library is provided in a vendor binary format or the library is provided in a simple tab‐separated format containing only the masses and assumed identities of peaks, but no metadata about source spectra, or key information such as the DDA analysis pipeline used including the FDR (False Discovery Rate) control applied, etc. Furthermore, most DIA datasets in the public domain do not include any spectral library at all. In these cases, while DIA raw data may be submitted, data reuse is challenging and full reproducibility becomes impossible. In the *Recommentations* section below, we return to this point to cover new developments planned in the coming years to improve this situation.

In this context of reusability, it is worth highlighting that at least there have been some initial attempts to develop guidelines for enabling the reproducibility of DIA published results [38], including the submission of all the relevant data including spectral libraries and related data to PX resources.

*Interoperability*. Raw DIA data is mostly as interoperable (or not) as regular DDA data, as discussed above. However, the current lack of interoperability for software in DIA is mostly driven by a lack of standardization or sharing of spectral libraries. The upshot is that there is very little independent re‐analysis of DIA data or attempts to reproduce published analyses with the same or different tools, with a few recent exceptions, for example ^[^ 39], where a pan‐human spectral library was used for the reanalysis, and therefore, reproducibility of the originally reported results was limited. This is bad for reproducibility of study outcomes, especially since it took many years for statistical methods to become robust and well embedded for controlling FDR in DDA proteomics. It is possible that with the use of inappropriate spectral libraries, and/or inappropriate FDR control, studies are being published reporting high proportions of incorrectly identified proteins.

### 1.4 Recommendations

We foresee an increasing flood of DIA proteomics studies in the literature, as many labs transition from DDA methods over to DIA on a large scale. We believe that there is some urgency to improve bioinformatics infrastructure and current practices in several areas for making DIA data more “FAIR,” to support making scientific outputs open, and to ensure the field develops high‐quality and reproducible analyses. These are our recommendations in different areas.

*Open data standard for spectral libraries*. There are different spectral library data formats, including among others the NIST (National Institute of Standards and Technology) MSP format, the SpectraST splib format, the Bibliospec blib format, and the SCIEX Peakview format. As mentioned above, while each of these formats performs adequately for storing the spectra and it is relatively easy to interconvert spectra between them, it is widely agreed that none of the formats captures important metadata about the collections of spectra themselves, and about the provenance of the spectra contained in the library. In order to advance the “FAIRness” of libraries, the field would greatly benefit from a community standardized spectral library format where the ability to encode complete metadata using CV terms was a central feature. The PSI is in the advanced stages of designing a new open spectral library format called mzSpecLib (https://github.com/HUPO‐PSI/mzSpecLib) where provenance and spectrum, analyte, and library metadata [40] are key components.

*Improvements in data provenance and sharing of spectral libraries in PX resources*. All labs publishing manuscripts that present DIA proteomics should perform data submissions to PX. Even if the submitted datasets are “Partial,” the sharing of spectral libraries used, ultimately ideally in mzSpecLib, but even within common open text‐based formats, would be a big improvement. This would facilitate other groups to benchmark different software packages, and even more importantly, to test, for example, whether using public libraries or in silico approaches can give better performance than experimentally matched libraries. Since so few experimentally matched libraries are publicly available, such comparisons are currently very limited. In due course, PX should then formalize mechanisms for submitting spectral libraries, so that they could be findable in an analogous manner to datasets, for example, by having their own identifiers, and including a clear link from library entries back to the originating individual spectra, by using, for example, the USI system.

It should also be noted that some DIA analysis tools produce in silico predicted spectral libraries “on‐the‐fly,” which are never written to a file. In this case, two alternatives would be possible. As a starting point, it would be mandatory to share all inputs needed for the software to run (e.g., sequence database), and provide the exact version of the software. This would require some extra work for the repositories, and it would make DIA datasets different depending on the tool and the type of analysis of approach used. In the medium term, once there is more experience working with these tools, software developers could consider enabling one option to export the predicted spectral libraries (and in fact most software tools already have this option).

Finally, it is worth mentioning this recommendation would also apply for relevant DDA datasets, that make use of spectral libraries in their analysis. The lack of spectral libraries has not been so critical so far for DDA approaches because the use of these approaches is quite small, when compared with sequence database‐based methods.

*Improved support for DIA data in PSI standards*. Many of the existing PSI data standards (e.g., mzML, mzIdentML, mzTab) were designed (at least originally) for DDA approaches. As DIA approaches mature, data standards and guidelines should be extended to better support DIA approaches. One recent example is the USI specification, which is centered around DDA fragmentation spectra and only includes an initial draft describing how to encode DIA spectra. Additionally, as noted above, there remains no widely accepted standard format for quantitative results in proteomics (for DDA or DIA data), and this needs to improve. However, it is important to highlight that, since DIA proteomics is still a relatively young field, new (improved) analysis software is being developed at a very high pace. As these tools often use novel approaches and/or methodology, data standardization is especially challenging.

There would be multiple advantages of having more tailored open data standards for DIA, considering some of the general benefits, as outlined above. One more concrete application would be the widespread availability of visualization software to enable manual inspection of peak groups for the peptide precursors identified. This functionality is offered at present by, for example, Skyline [41], but the availability of well‐adopted standards would enable that this functionality would be available in a much more widespread manner.

*Improved support for DIA datasets in PRIDE and other PX resources*. The original distinction between “Complete” and “Partial” datasets is tailored to DDA datasets. For DIA datasets a different categorization of datasets should be implemented, including criteria such as the format in which the spectral library is made available, and the compliance with some additional metadata annotation requirements. There is also the need to improve and standardize the linking between DIA datasets and the DDA ones that contained the source data for the generation of the libraries.

We would like to finish by highlighting that many of the points covered in this article do not only apply to proteomics. DIA techniques are also increasingly used in other fields where MS is used as an analytical technique (e.g., metabolomics, lipidomics, glycomics, etc). Many of the recommendations included here would indeed also be applicable to improve the “FAIRness” of data coming from those approaches.

Our teams will indeed contribute to these efforts via PSI and PX, but very importantly, these recommendations will also require the support of the proteomics community, for example, to take the extra effort to format and submit spectral libraries to PX. We would like to start further conversations in these areas, and welcome participation in PSI meetings and contributions to standards development discussions.

## CONFLICT OF INTEREST

The authors declare no conflict of interest.
